# Non-Invasive Pulse Wave Analysis in a Thrombus-Free Abdominal Aortic Aneurysm after Implantation of a Nitinol Aortic Endograft

**DOI:** 10.3389/fsurg.2015.00068

**Published:** 2016-01-11

**Authors:** Efstratios Georgakarakos, Christos Argyriou, George S. Georgiadis, Miltos K. Lazarides

**Affiliations:** ^1^Department of Vascular Surgery, University Hospital of Alexandroupolis, “Democritus” University of Thrace, Alexandroupolis, Greece

**Keywords:** abdominal aortic aneurysm, pulse wave velocity, pulse wave analysis, hemodynamics, augmentation index, endovascular aneurysm repair, arterial stiffness

## Abstract

Endovascular aneurysm repair has been associated with changes in arterial stiffness, as estimated by pulse wave velocity (PWV). This marker is influenced by the medical status of the patient, the elastic characteristics of the aneurysm wall, and the presence of intraluminal thrombus. Therefore, in order to delineate the influence of the endograft implantation in the early post-operative period, we conducted non-invasively pulse wave analysis in a male patient with an abdominal aortic aneurysm containing no intraluminal thrombus, unremarkable past medical history, and absence of peripheral arterial disease. The estimated parameters were the systolic and diastolic pressure calculated at the aortic level (central pressures), PWV, augmentation pressure (AP) and augmentation index (AI), pressure wave reflection magnitude (RM), and peripheral resistance. Central systolic and diastolic pressure decreased post-operatively. PWV showed subtle changes from 11.6 to 10.6 and 10.9 m/s at 1-week and 1-month, respectively. Accordingly, the AI decreased from 28 to 14% and continued to drop to 25%. The AP decreased gradually from 15 to 6 and 4 mmHg. The wave RM dropped from 68 to 52% at 1-month. Finally, the peripheral resistance dropped from 1.41 to 0.99 and 0.85 dyn × s × cm^−5^. Our example shows that the implantation of an aortic endograft can modify the pressure wave reflection over the aortic bifurcation without causing significant alterations in PWV.

## Introduction

Endovascular aneurysm repair (EVAR) is considered as the predominant treatment for Abdominal aortic aneurysms (AAA) in anatomically suitable candidates, conferring significantly lower perioperative morbidity and mortality compared to open repair (1). The implantation of an aortic stent-graft (SG) has been shown to influence the aortic stiffness, as estimated by arterial pulse wave velocity (PWV) (2). This effect is demonstrated quite early in the post-operative period, even in the first post-operative week, as shown by Lantelme et al. and Takeda et al. (3, 4).

The majority of recent reports have focused solely on the influence of EVAR on PWV as a surrogate of aortic stiffness. However, the impact of EVAR on stiffness can be influenced by many factors, such as the amount, distribution, and elasticity of the intaraluminal thrombus, the intrinsic compliance of the AAA wall, and the stiffness characteristics of the SG (5–8). Additionally, factors such as arterial hypertension, smoking, chronic kidney disease, diabetes mellitus, and peripheral arterial disease are known to increase the arterial stiffness of the patient (9).

In order to estimate the net effect of the implantation of a SG in AAA, we studied a representative example of an AAA with no intraluminal thrombus in a patient presenting none of the factors mentioned above, and we assumed that any hemodynamic change would be attributed to the sole implantation of the SG itself. Since there is evidence that EVAR may affect the magnitude of reflected pressure waves with potential implication on cardiac function (10, 11), we estimated non-invasively various indices of arterial stiffness as well as parameters related to pressure wave reflection, listed below.

## Case Presentation

**Figure 1:**
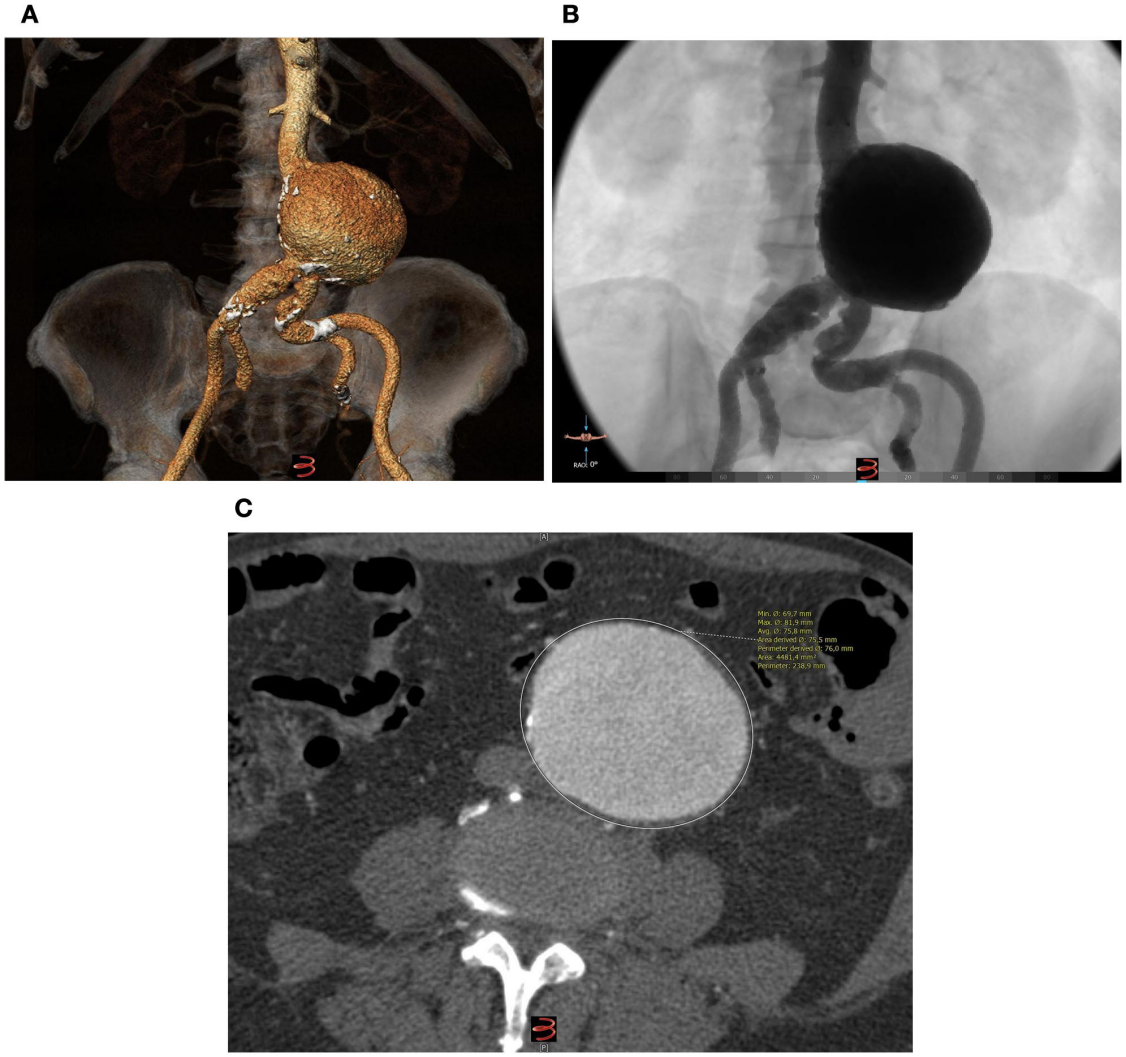
**(A)** Pre-operative 3D reconstruction (3Mentio Medical Imaging B.V., Bilthoven, The Netherlands) of the 7.5-cm abdominal aortic aneurysm. The aneurysm contains no intraluminal thrombus at all, as can be seen in the coronal **(B)** and axial plane **(C)** of the computed-tomography angiography.

An 85-year-old male patient was admitted to our Vascular Department with a saccular infrarenal AAA of 7.5 cm maximum diameter. The infrarenal neck was cylindrical, presenting no angulation whereas its diameter and length were 21 and 33 mm, respectively. The distance from the lowermost (left) renal artery to the aortic bifurcation was 105 mm. The left common iliac artery presented with 90° angulation (Figure 1A). The patient was non-smoker and presented no obesity whereas his medical history revealed no hypertension, diabetes mellitus, renal insufficiency, or peripheral arterial disease (ankle-brachial index 1.1). No coronary disease or atrial fibrillation was reported. Notably, the AAA presented no intraluminal thrombus at all (Figures 1B,C).

**Figure 2:**
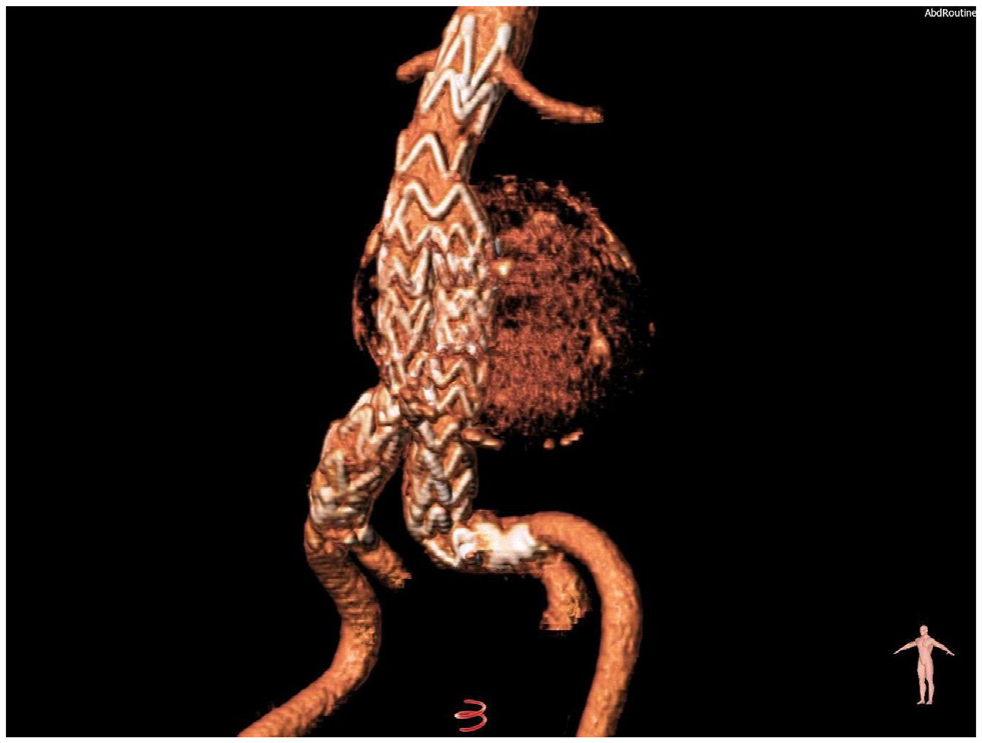
**Volume-rendering reconstruction of the aneurysm after the implantation of a modular nitinol-based endograft (Bolton Medical, Barcelona, Spain)**.

Through open femoral artery exposure under spinal anesthesia, we successfully implanted the Treovance (Bolton Medical, Barcelona, Spain) aortic SG, a modular endovascular graft composed of a series of self-expanding serpentine Nitinol stents sutured to tightly woven polyester vascular graft fabric (12, 13). The chosen proximal diameter of the endograft was 24 mm (corresponding to 10% oversizing) with a main body length of 80 mm (Figure 2).

**Figure 3:**
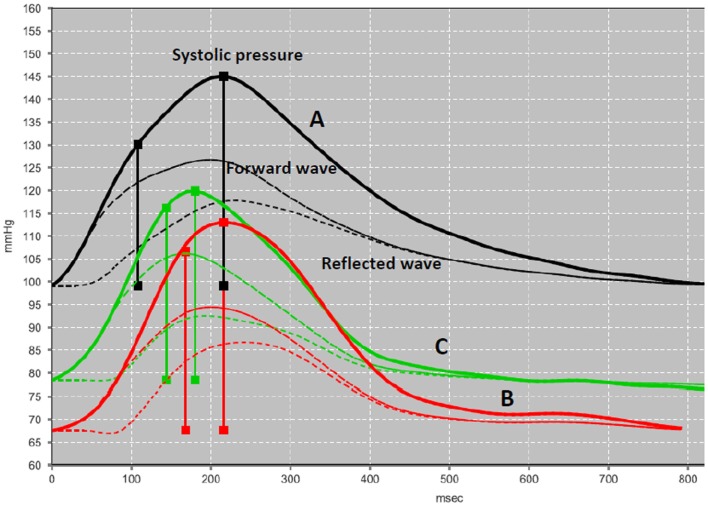
**Pulse wave analysis**. A validated software (ARCSolver) reconstructs the central pulse wave and calculates the amplitudes of forward and reflection waves. The pressure curves correspond to the preoperative state (A), first post-operative week (B), and first post-operative month (C).

A brachial cuff-based automatic oscillometric device (Mobil-O-Graph, IEM, Stolberg, Germany) was used to perform pulse wave analysis using a mathematical transformation (ARCSolver, Austrian Institute of Technology, Vienna, Austria) to provide the brachial cuff waveform readings, the reconstruction of the central pulse, and the wave separation analysis (14, 15). The quantification of pulse wave reflections (Figure 3) focuses on estimation of: (i) the central aortic systolic pressure and (ii) its augmentation through reflections in the vasculature. Moreover, the wave separation analysis quantifies the total amount of arterial wave reflection considering both aortic pulse and flow waves (16, 17). Further details of the functional principles of the device have been extensively analyzed, elsewhere (18). The calculated hemodynamic parameters are listed below.

### Calculated Hemodynamic Parameters

Central systolic, diastolic, and pulse pressure (cSyst, cDiast, cPP), peripheral resistance (PR), and cardiac index (CI) were assessed pre-operatively and post-operatively. Moreover, the following parameters were calculated, as surrogates of wave reflections and arterial stiffness:

- Augmentation Index (AI) adjusted at heart rate 75 beats/min (AI@75) reflects the increase of aortic systolic BP due to wave reflections.
- Augmentation Pressure (AP) is determined by the difference of the pressure at second inflection point minus the pressure at first inflection point of the systolic part of pressure wave. The first inflection point is indicative of the arrival of reflected waves at ascending aorta.
- Reflection magnitude (RM) is defined as the ratio of the amplitude of the backward (reflected) wave and the forward (incident) wave. The amplitude of the forward and the reflected pressure wave was quantified using wave separation analysis, which was performed by the ARCSolver method. This method uses pressure and flow waves to perform frequency domain-based calculations to derive the amplitudes of the forward and backward traveling waves as previously described (14).
- PWV is considered as a valid and clinically feasible surrogate of aortic stiffness, correlating with cardiovascular disease. It is estimated from the time difference between the forward and reflected waves (17).

Measurements were performed 1 day before surgery, at 1 week, and at the end of the first post-operative month. All measurements were conducted at rest, after abstention from caffeine intake for at least 12 h. Written informed consent was given by the patient for the measurements and use of the data, in accordance with the Declaration of Helsinki.

## Results

Table 1 demonstrates the hemodynamic measurements before and after EVAR, as described above. There was a decrease of cSystolic and cDiastolic pressure whereas the cPP remained constant. The preoperative PWV was 11.6 m/s and remained quite constant to 10.6 and 10.9 by the end of the first post-operative week and first post-operative month, respectively. AI@75 decreased significantly by half (from 28 to 14) at 1 week and dropped further to 25% of the preoperative value at 1 month. Accordingly, the AP decreased gradually from 15 to 6 and 4 mmHg at 1 month. Similarly, the RM dropped from 68 to 52% at first month. Interestingly, the CI showed a continuous increase from 2.9 pre-operatively to 3.1 l/min × 1/m^2^ at first week and further to 4.3 l/min × 1/m^2^ at 1 month. Finally, the PR dropped from 1.41 preoperatively to 0.99 and 0.85 dyn s cm^−5^, respectively.

**Table 1:** **Values of hemodynamic parameters between preoperatively and post-operatively**.

	Preoperatively	1-Week	1-Month
cSystolic	145	113	120
cDiastolic	99	68	78
cPP	46	45	42
PWV	11.6	10.6	10.9
AI@75	28	14	7
RM	68	76	52
AP	15	6	4
CI	2.9	3.1	4.3
PR	1.41	0.99	0.85

*PWV, pulse wave velocity; AI@75, augmentation index (adjusted for a heart rate of 75 beats/min); RM (%), reflection magnitude; AP, augmentation pressure; CI, cardiac index (l/min × 1/m^2^); PR, Peripheral Resistance (dyn s cm^−5^)*.

## Discussion

Pulse wave velocity along with AI@75 comprises useful surrogates of the arterial compliance and, additionally, a marker of therapeutic (pharmaceutical or interventional) interventions in the arterial tree (19, 20). As the arterial compliance declines with age, the wave speed increases and, consequently, reflected pressure waves tend to return earlier in older people (17). Arterial stiffness is associated with left ventricular hypertrophy, which has been linked to increased risk of atherosclerotic heart disease, myocardial infarction, and stroke (19). Elevated PWV comprises a significant marker and predictor of cardiovascular risk in hypertensive patients (19). Thus, patients with ischemic heart disease who undergo EVAR might be at a higher cardiovascular risk after the SG implantation than preoperatively. Interestingly, in the EVAR-1 trial, an increase in cardiovascular event and death rates were higher during the first 6 months after EVAR than during the subsequent follow-up intervals (21). Therefore, it is imperative to investigate the causative association between the implantation of a SG and the imposed mechanical/hemodynamic alterations (PWV, arterial impedance, compliance, wave reflection). PWV and AI@75 present elevated values in patients with AAA (11). Such patients have a significantly higher 5-year incidence of adverse cardiovascular events compared with the expected survival of a matched population (22). In addition to that, in the presence of significant coronary disease, the survival of these patients tends to be worse (22).

It has been suggested that aortic stiffness can be influenced by the implantation of a stiff SG within the aorta, as estimated with PWV. However, in order to draw conclusions and interpret the magnitude or the reason of alteration of aortic stiffness in such studies, one should take into serious consideration the significant variation in results between patients with AAA as well as the discrepancies in applied measurement methodologies, as documented by Lantelme et al. (3). (23, 24). Moreover, since PWV is proportional to the square root of aortic stiffness and inversely proportional to the square root of aortic radius, a question remains regarding whether an increase in PWV after EVAR reflects actually the effect of restoration of a uniform and smaller cross-sectional area along the abdominal aorta rather than indicating a valid post-operative increase in aortic stiffness (25). Therefore, additional hemodynamic parameters such as AI@75 and pulse wave reflection magnitude should be taken into consideration in order to comprehend the practical meaning of EVAR on arterial stiffness.

Our case presents some unique features that help us focus on the sole effect of SG implantation on the circulatory system; the patient was a normotensive non-smoker, with no history of diabetes mellitus, renal failure, or peripheral arteriopathy; in other words, there were no known factors other than age and the presence of AAA contributing to the increased aortic stiffness. Moreover, the AAA had no intraluminal thrombus at all, which is considered as an inhomogenous material whose structural and mechanical properties are difficult to predict especially with respect to the influence on aortic stiffness. Therefore, the elevated PWV – accommodated for the patient’s age and gender – can be attributed to the sole effect of the aneurysm wall, in accordance with previous reports. Moreover, any post-operative changes would be attributed to the implantation of the SG.

The PWV remained quite constant in the immediate post-operative period. On the other hand, the pressure wave analysis revealed significant changes in the transmission of the pressure wave after the implantation of the endograft. PWV expresses arterial stiffness while AI@75 indicates small artery elasticity. The decrease of AI@75 and RM implies that the peripheral vasodilation, as documented by reduction in PR, can modify the amplitude of the backward and the forward wave (reflection magnitude) and delay the pressure wave reflection toward the ascending aorta. The deceleration of the reflected wave is also mirrored in the magnitude of the AP, which is declined by more than half. This improvement in AI@75 and PR denotes a post-EVAR peripheral arteriolar adaptation that has never been marked before.

As previously stressed, the ideal parameters or our examples help elucidate some crucial points. First of all, it is evident that the restoration of a smaller diameter flow lumen does not coincide with a post-operative increase in aortic stiffness. This may be explained by previous studies, which showed the contribution of the distal aorta to the total arterial compliance to be the least, compared to central segments of the aorta (26, 27). It is rather an AAA-induced disturbed pattern of pressure wave reflection that is pre-operatively expressed as elevated PWV and AI@75. More interestingly, recruitment of an efficient peripheral arteriolar adaptation mechanism is thought to reserve a role much more crucial as previously presumed. It seems that the implantation of an abdominal SG restores a normal geometrical flow pattern that triggers the aforementioned beneficial mechanisms to counteract the potential effect of increased stiffness and decreased compliance imposed by some SG. Future studies should evaluate the possible implications of our findings, such as an improved myocardial performance due to decreased afterload with consequent reduction in the need of antihypertensive regimens and decrease in the incidence of cardiovascular adverse events post-interventionally (28).

Our example documents the initial steps of the EVAR–central circulation interaction: changing the geometry of the lower aortic conduit and affecting precisely the pulse wave reflection. Introducing this pathophysiological aspect of EVAR may alarm both the clinicians and bioengineers to adapt or develop materials that would comply better to patients’ physiology, especially of those with moderate or severe myocardial performance. Delineating the potential modes of action of a particular therapeutic agent at the initial steps (and later) provides a better understanding of its pathophysiologic influences and, accordingly, of ways to improve it. Moreover, this hemodynamic approach to EVAR mechanics could be useful to evaluate the short- or long-term impact of totally novel endovascular therapeutics, AAA sealing (e.g., Nellix^®^ endovascular aneurysm sealing system, Endologix, Inc., Irvine, CA, USA) where the entire sac volume is filled with a solidified polymer, rendering two SGs as the new flow pathway and relocation, by definition, the actual site of flow bifurcation (flow divider) more centrally (29). Moreover, the quite stiff endoskeleton of AFX (Endologix, Irvine, CA, USA), which accommodates directly onto the aortic bifurcation may also have a different effect on central hemodynamics (30). In other words, our method of demonstration and hypothesis may provide useful comparisons in future studies with respect to totally different designs of endografts.

It should be stressed out that the inclusion of a single – yet representative – case does not allow drawing certain conclusions and detecting clinical valuable comparisons. However, it describes an insightful way of investigating the potential influence of EVAR on hemodynamics, setting at the same time standards and requirements for an up-coming larger study with greater follow-up.

To conclude, our case study implies that the implantation of an aortic SG in patients with AAA may modify the pressure wave reflection without necessarily causing significant alterations in PWV. Consequently, further studies on myocardial performance in large patient populations are expected to delineate the precise influence of different designs of aortic endografts on the cardiovascular impairment as well on the survival of AAA patients.

## Author Contributions

Dr. EG: conception and design of research, data collection, interpretation of results, and writing the article. Dr. CA: data collection, interpretation of results, and writing the article. Dr. GG: critical revision of the article. Prof. ML: overall responsibility.

## Conflict of Interest Statement

The authors declare that the research was conducted in the absence of any commercial or financial relationships that could be construed as a potential conflict of interest.
